# Ultra-fast artificial neuron: generation of picosecond-duration spikes in a current-driven antiferromagnetic auto-oscillator

**DOI:** 10.1038/s41598-018-33697-0

**Published:** 2018-10-24

**Authors:** Roman Khymyn, Ivan Lisenkov, James Voorheis, Olga Sulymenko, Oleksandr Prokopenko, Vasil Tiberkevich, Johan Akerman, Andrei Slavin

**Affiliations:** 1 Department of Physics, University of Gothenburg, 41296 Gothenburg, Sweden; 2 Department of Electrical Engineering and Computer Science, Oregon State University, Corvallis, OR 97331 USA; 3 Department of Physics, Oakland University, Rochester, MI 48309 USA; 4 Faculty of Radiophysics, Electronics and Computer Systems, Taras Shevchenko National University of Kyiv, Kyiv, 01601 Ukraine; 5 Present Address: Electrical and Computer Engineering Department, Northeastern University, Boston, MA 02464 USA; 6 Material Physics, School of Engineering Sciences, KTH Royal Institute of Technology, 16440 Kista, Sweden

## Abstract

We demonstrate analytically and numerically, that a thin film of an antiferromagnetic (AFM) material, having biaxial magnetic anisotropy and being driven by an external spin-transfer torque signal, can be used for the generation of ultra-short “Dirac-delta-like” spikes. The duration of the generated spikes is several picoseconds for typical AFM materials and is determined by the inplane magnetic anisotropy and the effective damping of the AFM material. The generated output signal can consist of a single spike or a discrete group of spikes (“bursting”), which depends on the repetition (clock) rate, amplitude, and shape of the external control signal. The spike generation occurs only when the amplitude of the control signal exceeds a certain threshold, similar to the action of a biological neuron in response to an external stimulus. The “threshold” behavior of the proposed AFM spike generator makes possible its application not only in the traditional microwave signal processing but also in the future neuromorphic signal processing circuits working at clock frequencies of tens of gigahertz.

## Introduction

Generators of short in the time domain “Dirac-delta-like” pulses are widely used in modern electronics and optics. The most obvious applications of such generators are for the formation of trigger sequences, pulse-density modulation (PDM) of signals, and other signal processing purposes. In PDM the amplitude of an analog input signal is encoded by the relative repetition rate of the generated pulses. A train of “Dirac-delta-like” pulses with a constant repetition rate forms a Fourier image of equidistant sharp peaks in the frequency domain, which is known as a frequency “comb”. Generators of frequency-“combs” are used for dense frequency-division multiplexing (DFDM) in electronics, and dense linewidth-division multiplexing (DLDM) in optics to exploit the full bandwidth of the data transmission lines. In this approach, the generators of the carrier frequencies can be locked to a corresponding frequency of the “comb”. Thus, one of the key characteristics of signal processing devices using the “comb” generators is the bandwidth of the frequency-“comb” generator, which is limited by the duration of a single pulse.

A similar type of pulse-encoded signals is used in nervous systems of biological objects, where response of a neuron to an input stimulus is a single spike, or a train of spikes with a certain sequence frequency, which is called an action potential in the cell biology^1^. Therefore, the modern concepts of neuromorphic computing and signal processing include spike generators as a mandatory element of their architecture^2,3^. Another peculiarity of a nervous system is the neuron’s threshold behavior, in a sense, that a neuron generates a response *only* when the input stimulus is above a certain critical value (threshold). The non-linear behavior of neurons allows massively parallel signal processing in biological neural networks with very low power consumption. While a lot of effort has been made to replicate the neuron behavior using electronic systems^4^, it is extremely difficult to create functioning electronic circuits with the numbers of neurons (10^11^) and interconnecting synapses (10^13^) comparable to the numbers existing in a human brain^5^. At the same time, biological neuron networks are rather slow, due to the relatively low speed of the natural biological processes. Thus, it is interesting and perspective to try to develop ultra-fast artificial neurons, anticipating that the increased operation speed will compensate the smaller number of elements in artificial neuromorphic networks (compared to human brain) in performing comparable information processing tasks.

Electronic frequency-“comb” generators, usually, employ the modern CMOS technology, and can have a compact design, but operate at relatively low frequencies, and, therefore, have a relatively low frequency bandwidth (< 50 GHz)^6^. The optical comb generators offering microwave frequency spacing, based on the phase modulation in Fabry-Perot cavities^7^, multi-frequency lasers^8^, Brillouin-enhanced fiber lasers^9^ and phase modulation within an amplified fiber loop^10^ can have a substantially wider frequency span (> 100 GHz), but are rather complex devices, incompatible with the existing on-chip technology.

Spin-torque nano-ocillators (STNO) and spin-Hall oscillators (SHO) based on ferromagnetic (FM) materials^11–15^ are of a high interest for modern spintronics as tunable nano-scale generators of microwave signals, and, in principle, can be used as pulse generators, but their typical response time is determined by the frequency of the ferromagnetic resonance, and is limited to hundreds of picoseconds by the practically achievable magnitudes of the local bias magnetic field.

Recently, however, it has been proposed to use AFM materials as active layers of SHOs due to their ability to operate at higher frequencies, up to the THz range^16–21^. In an AFM-based SHO, the spin current ${j}_{s}^{in}$ created by the spin-Hall effect (SHE) in the adjacent heavy metal layer traversed by a direct electric current *j*, induces a torque on the Neel vector **l** of the AFM. If the spin polarization **p** of the driving current in AFM is perpendicular to the equilibrium orientation of the Neel vector **l**_0_, the Neel vector starts to rotate in the plane perpendicular to the vector **p**^16,20,21^.

The extraction of an ac signal by the inverse SHE (ISHE) in the adjacent layer of a heavy metal is, however, non-trivial, because it requires the motion of the Neel vector that is non-uniform in time. Several approaches were proposed to solve this problem^17,20,21^. For instance, it was shown in ref.^20^, that a non-uniform rotation of the Neel vector can be achieved in AFM materials with bi-axial type of anisotropy (e.g., NiO), where additional in-plane anisotropy creates an effective potential profile for the rotating Neel vector. The output signal of the AFM generators based on this mechanism, is however, a simple harmonic (sinusoidal) oscillation.

**Figure 1:**
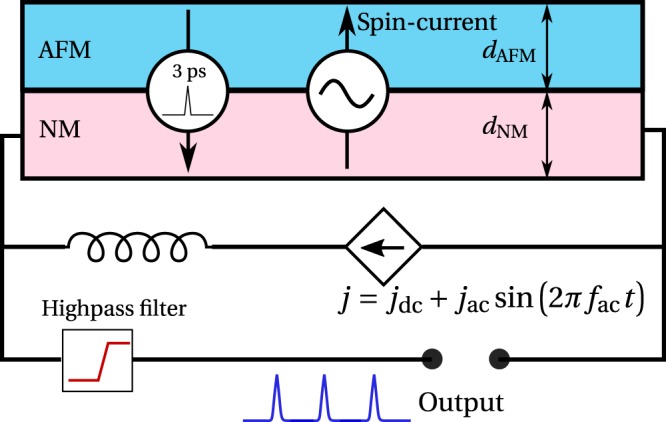
Principal operational scheme of a spintronic generator of ultra short pulses (spikes). A thin antiferromagnetic (AFM) layer is covered by a layer of a normal metal (NM). The NM layer is driven by an ac-modulated electrical current. The output signal is received through a high-pass filter allowing only the frequencies higher than the modulation frequency to get out.

Here, we propose a design of an AFM-based spin-Hall auto-oscillator, capable of generating controlled sequences of ultrashort pulses with a typical pulse duration of a few picoseconds. The proposed generator is based on a layered structure consisting of a current-driven layer of a normal metal (NM) with a strong spin orbit-coupling, and an antiferromagnetic (AFM) layer with a biaxial magnetic anisotropy. Spin current (${j}_{s}^{in}$) created by the spin-Hall effect (SHE) in the NM and flowing into a thin AFM film creates a torque on the sublattice magnetizations of the AFM, which leads to a rapid switch of their orientation. This switch, in its turn, creates a short pulse of the spin current flowing back to the NM layer (${j}_{s}^{out}$), where it can be converted into an electrical signal by the inverse spin-Hall effect (ISHE), see Fig. 1.

The minimum duration of a short pulse is limited by a characteristics time of the current-induced AFM sublattice reorientation process. For a relative strong magnetic damping, this reorientation time is proportional to the effective Gilbert damping constant and inversely proportional to the magnitude of the in-plane magnetic anisotropy field in the AFM material (see Eq. (2) below). Since the effective Gilbert damping constant in sandwiched AFM/NM structures is mostly determined by the spin-pumping^22^, it can be controlled, and the achievable reorientation time can be of the order of several picoseconds for typical AFM/NM bilayers, such as nickel oxide (NiO) and Pt. The pulse repetition rate in the proposed generator of ultrashort pulses can be easily controlled by the frequency of the input electrical current, and can reach values exceeding 100 GHz. The short duration of a single generated pulse, also, defines a wide frequency span of the generated frequency comb −200 GHz at −10 dB for NiO.

The generation of short spikes in the above proposed AFM-based pulse generator happens to be a *threshold process* with respect to the amplitude of the input driving electric current, which gives us a possibility to consider such an oscillator as an *ultra-fast analogue of a biological neuron*. Moreover, the complex behavior of a neuron, such as generation of discrete groups of spikes (bursting^3^), can be also achieved in the proposed AFM generator for certain parameters of the driving ac electric signal.

## Results

We consider theoretically a thin film of an AFM material, which has two magnetic sublattices with magnetizations **M**_1_ and **M**_2_. The film exhibits a biaxial magnetic anisotropy: the anisotropy easy plane is perpendicular to the axis **n**_*h*_, and its effective magnetic field is *H*_*h*_, while the anisotropy easy axis is directed along the vector **n**_*e*_, and its effective magnetic field is *H*_*e*_^20^. We shall also assume, that the polarization of the spin current flowing into the AFM layer is directed along the hard axis of the AFM anisotropy ${\bf{p}}\parallel {{\bf{n}}}_{h}$.

The spin dynamics of the AFM with bi-axial anisotropy under the action of an external spin torque is described by the set of two coupled Landau-Lifshitz equations for the magnetization of each of the AFM sublattices **M**_**1**_ and **M**_**2**_, see Methods section for details. We study the AFM dynamics under the action of such a STT which varies in time. The torque is created by SHE in the adjacent Pt layer, see Fig. 1, due to electrical current *j* flowing in the Pt layer. We solved numerically the set of coupled Landau-Lifshitz equations for **M**_1_ and **M**_1_ with the applied current (see Eqs (11 and 12) in^20^ and Methods section for details).

This solution was performed for a time-modulated electric current *j* = *j*_*dc*_ + *j*_*ac*_sin (2*πf*_*ac*_*t*) in a wide range of the following experimentally controllable parameters of the driving signal and the AFM material: *j*_*dc*_ - the amplitude of the dc component of the applied current, *j*_*ac*_, *f*_*ac*_ - the amplitude and frequency of the ac component of the applied current, and the effective damping constant *α*_*eff*_ of the AFM material. In our numerical simulations we assumed a Pt NM layer and the AFM layer made of NiO. We took all the material parameters from ref.^20^, except for *α*_*eff*_.

**Figure 2:**
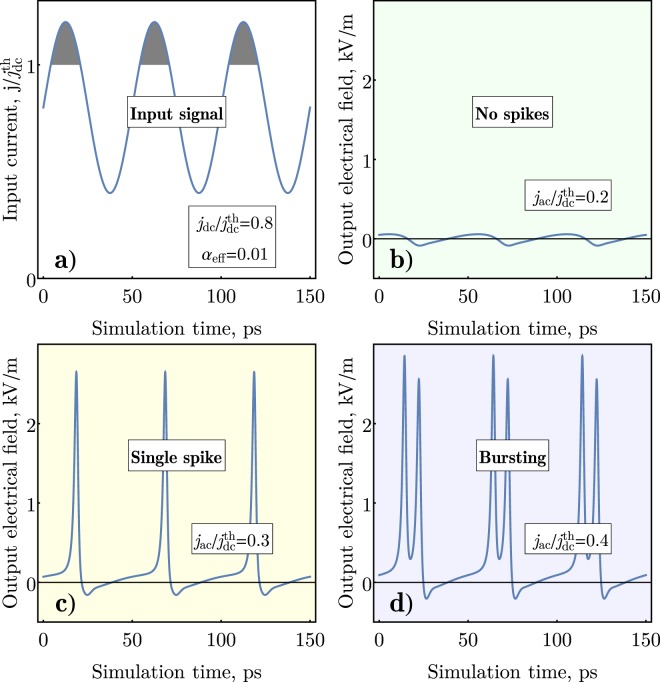
Numerical simulation of different regimes of spike generation in the AFM SHO: (**a**) Shape of the combined (dc + ac) input driving current; (**b**) Regime of no generation (“low” value of the ac current *j*_*ac*_); (**c**) Generation of a single output spike during each period of the driving ac current (“moderate” values of the ac current *j*_*ac*_); (**d**) Generation of a discrete group of spikes (bursting) during each period of the AC current (“high” values of the ac current *j*_*ac*_).

**Figure 3:**
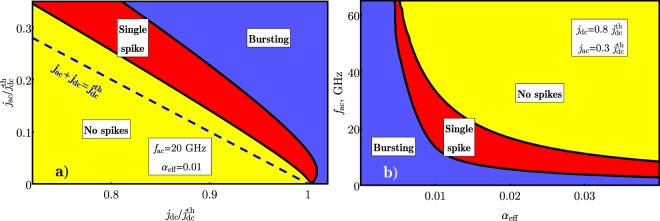
“Phase diagrams” of the spike generation regimes for different values of the experimentally controllable parameters of the driving current *j*_*dc*_, *j*_*ac*_, *f*_*ac*_ and the AFM material dissipation *α*_*eff*_: (**a**) “Phase diagram” on the plane ${j}_{ac}\,/\,{j}_{dc}^{th}$ vs. ${j}_{dc}\,/\,{j}_{dc}^{th}$ at the fixed value of the driving ac frequency *f*_*ac*_ = 20 GHz and *α*_*eff*_ = 0.01; (**b**) “Phase diagram” on the plane *f*_*ac*_ vs. *α*_*eff*_ at the fixed values of ${j}_{dc}\,/\,{j}_{dc}^{th}=0.8$ and ${j}_{ac}\,/\,{j}_{dc}^{th}=0.3$.

The examples of the input and output signals of the proposed AFM spike generator are shown on Fig. 2. Figure 2(a) shows the input driving current with a dc component fixed below the threshold ${j}_{dc} < {j}_{dc}^{th}$. At the low values of the ac current amplitude *j*_*ac*_ the generator does not produce a significant output signal (Fig. 2(b)). With the increase of the *j*_*ac*_, at some point when the combined amplitude exceeds a generation threshold ${j}_{dc}+{j}_{ac}={j}_{1}^{th}$, the device generates a single sharp spike of a significant amplitude during each period of applied ac current, (Fig. 2(c)), i. e. a periodic sequence of spikes (or a temporal *“comb”*) is generated. For the parameters used in our numerical simulation (see Fig. 2) the duration of the numerically simulated spike Δ*t* = 2.4ps is close to the value analytically estimated using Eq. (2). The spike generation has a well-defined threshold on *j*_*ac*_, which is similar to the response of a biological neuron to an external stimulus, and the shape of the spike produced by the AFM generator also replicates a typical shape of a spike produced by a biological neuron^1^. With the further increase of the amplitude of the ac current above the threshold, the Neel vector could switch two (2*π* rotation) or more (*πn* rotation) times per one period of the driving ac current (see Fig. 2(d). Such a behavior is known for biological neurons as “bursting”^3^. The generation of “bursts” with a desired number *n* of spikes, however, requires a fine tuning of the ac amplitude, because the range of the required ac current amplitude *j*_*ac*_ rapidly decreases with the increase of *n*. Thus, below we categorize the reactions of our artificial AFM “neuron” to the external “stimulus” into “no spikes”, “single spike”, or “bursting” regimes, independently of the number of spikes generated in a “burst” (see Fig. 3).

The “phase diagrams” of the spike generation regimes in an AFM auto-oscillator driven by a combined (dc + ac) control signal are shown on Fig. 3. Two “phase diagrams” are presented: the “phase diagram” on the plane *j*_*ac*_ vs. *j*_*dc*_ at the fixed values of the driving ac frequency *f*_*ac*_ = 20 GHz and the AFM material damping *α*_*eff*_ = 0.01 (see Fig. 3(a)), and the “phase diagram” on the plane *f*_*ac*_ vs. *α*_*eff*_ at the fixed values of ${j}_{dc}=0.8\,{j}_{dc}^{th}$ and ${j}_{ac}=0.3\,{j}_{dc}^{th}$ (see Fig. 3(b)).

## Discussion

The above described numerical solution quantitatively describes the dynamics in the AFM film, however, does not allow qualitative analysis. To render an analytic analysis we use so-called *σ*-model for antiferromagnets^16,23,24^. In the low frequency limit (i.e. when the frequency of oscillations is much less than the exchange frequency ≈30 THz) for the considered geometry of an AFM SHO this set of equations can be reduced to the single equation describing the dynamics of the rotation angle *ϕ* of the Neel vector **l** = (**M**_1_ − **M**_2_)/2*M*_*s*_ in the easy plane of the AFM^20^. In terms of the angle *ϕ*, which is is measured from the easy axis **n**_*e*_ (**l**⋅**n**_*e*_ = cos*ϕ*), the equation for the Neel vector dynamics has the following form^20^:

1 $$\frac{1}{{\omega }_{ex}}\ddot{\varphi }+{\alpha }_{{\rm{eff}}}\dot{\varphi }+\frac{{\omega }_{e}}{2}\,\sin \,2\varphi =\sigma {j}_{e}(t),$$

where *ω*_*ex*_ = *γH*_*ex*_ is the exchange frequency and *ω*_*e*_ = *γH*_*e*_. The term in the right-hand-side part of Eq. (1) describes the spin torque created by the electrical current *j*_*e*_(*t*) flowing in NM layer. Coefficient *σ* expresses the spin-current generated by electric current *j*_*e*_ in the frequency units^20,25^. The effective damping parameter *α*_e*ff*_ = *α*_0_ + *α*_*SP*_ includes both the intrinsic Gilbert damping constant *α*_0_ and the additional magnetic losses due to the spin pumping from the adjacent layer of the normal metal *α*_*SP*_^22^. The losses due to the spin pumping depend on the thickness *d*_*AFM*_ of the AFM layer (${\alpha }_{SP} \sim 1/{d}_{AFM}$), and, therefore, can be adjusted in a certain range by a proper design of the geometric parameters of the AFM SHO.

**Figure 4:**
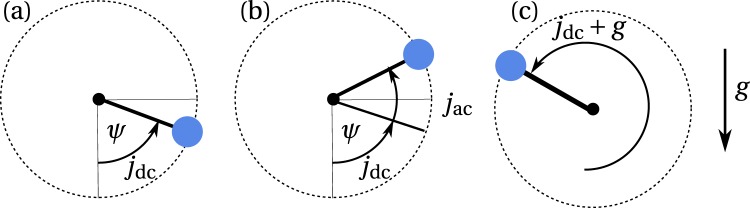
The dynamics of a physical pendulum as an analogue to the dynamics of the AFM generator. Angle *ψ* of the pendulum corresponds to *ϕ*/2 angle of the Neel vector in the AFM: (**a**) “below threshold” torque *j*_dc_ lifts the pendulum from the ground state; (**b**) additional controllable torque *j*_ac_ transfers the oscillator through the threshold; (**c**) fast motion of the pendulum, which corresponds to the AFM sublattice reorientation.

It is really remarkable, that the spin dynamics of an AFM oscillator described by Eq. (1) is mathematically analogous to the dynamics of a physical pendulum in a gravitational potential under the action of an external torque *σj*_*e*_(*t*). In this analogy the magnetic anisotropy in the AFM layer plays the role of a gravitational field *g*, Gilbert damping plays the role of a friction, and the inverse of the exchange frequency plays the role of the pendulum inertial mass. For the qualitative analysis of *over-damped* oscillator ($2{\alpha }_{{\rm{eff}}} > \pi \sigma {j}_{e}/\sqrt{{\omega }_{e}{\omega }_{ex}}$) one can neglect the first inertial term in Eq. (1). Since the gravity is a directional field and the magnetic anisotropy is bi-directional, to make the full analogy we have to replace the angle of the Neel vector *ϕ* with the angle of the pendulum *ψ* as 2*ϕ* = *ψ*, see Fig. 4(a).

In the absence of the external torque *j*_*e*_ = 0 the “AFM pendulum” is in a ground state, which defines the energy minimum *ψ* = *ϕ* = 0. A small, steady in time driving torque lifts the “pendulum” to a tilt angle *ψ*_0_ = arcsin(2*σj*_*dc*_/*ω*_*e*_). This happens when the dc current is flowing in the NM layer *j*_*e*_ = *j*_*dc*_ = c*onst*. The maximum value of the tilt angle at a stationary state of the pendulum is ${\psi }_{0}^{max}=\pi /2$, because at this angle the returning torque from the “gravity” potential is maximized, and, if the torque overcomes this threshold $\sigma {j}_{dc} > \sigma {j}_{dc}^{th}={\omega }_{e}/2$, the pendulum undergoes an infinite rotational motion^20^.

Now, let us consider the dynamics of the pendulum when in addition to a steady in time torque *σj*_*dc*_ some additional torque *σj*_*t*_ is applied for a short period of time. In the initial situation the pendulum remains tiled with an angle *ϕ*_0_. After an additional torque *j*_*t*_ is turned on, the angle *ϕ* starts to increase. If ${j}_{dc}+{j}_{t} > {j}_{dc}^{th}$, the pendulum overcomes the threshold angle *ψ* > *π*/2. At this point the torque created by the *j*_*dc*_ alone is sufficient to continue the rotation of the pendulum, and, therefore, one could turn off the additional current *j*_*t*_. Importantly, when *ψ* > *π* the returning force, coming from the “gravitational” (or anisotropy) potential, is now assisting the torque *j*_*dc*_, which results in a very fast acceleration of the pendulum in the region *π* < *ψ* < 2*π*. If the damping is sufficiently large to stop the infinite rotation ($2{\alpha }_{{\rm{eff}}} > \pi \sigma {j}_{dc}\,/\,\sqrt{{\omega }_{e}{\omega }_{ex}}$), the pendulum will relax to a new stationary point *ϕ*_0_ + *π*, which corresponds to the switching of the magnetization sublattices **M**_1_ and **M**_2_ to an opposite direction. Note, that we are interested in the case of relatively small values of the control current *j*_*t*_, that requires the bias current *j*_*dc*_ to be close to the threshold value (${j}_{dc}\simeq {j}_{dc}^{th}$, and the value of the effective damping in the AFM material to be rather large $4{\alpha }_{e{\rm{ff}}} > \pi \sqrt{{\omega }_{e}/{\omega }_{ex}}$.

The electrical field in the NM layer, produced by the back spin-pumping through the ISHE, is proportional to the angular velocity of the Neel vector: *E* = *κ*$\dot{\varphi }$. The pendulum reaches maximum velocity $\dot{\varphi }$_*max*_ (which determines the spike amplitude) at $\ddot{\varphi }$ = 0, and for the above described scenario of the spike generation one gets from Eq. (1): $\dot{\varphi }$_*max*_ = (*σj*_*dc*_ + *ω*_*e*_/2)/*α*, or $\dot{\varphi }$_*max*_ = *ω*_*e*_/*α* when *j*_*dc*_ is close to the threshold value (${j}_{dc}\simeq {j}_{dc}^{th}$). Thus, the Neel vector **l** rotates through the angle *π* during one spike, and one can introduce a characteristic time:

2 $${\rm{\Delta }}t=\pi {\alpha }_{eff}/{\omega }_{e},$$

which defines the duration of the spike. For *α*_*eff*_ = 0.01 and *ω*_*e*_/2*π* = 1.75 GHz, which are typical for the AFM NiO^26^, one gets Δ*t* = 2.85ps.

To achieve the continuous generation of a sequence of spikes, having a “comb”-like spectrum in the frequency domain, the additional current *j*_*t*_ in the NM layer should be replaced by a periodically modulated control signal:

3 $${j}_{e}={j}_{dc}+{j}_{ac}\,\sin \,2\pi {f}_{ac}t.$$

As one can see from Fig. 3(a) the threshold for the spike sequence generation ${j}_{ac}={j}_{1}^{th}$ lays above the line ${j}_{ac}+{j}_{dc}={j}_{dc}^{th}$, because the applied ac current, after overcoming the potential barrier caused by the perpendicular anisotropy in the AFM “easy” plane, must produce a sufficient work against the effective damping (see Fig. 4). The work produced by the ac current depends on the duration of its action, and, therefore, on the ac current frequency *f*_*ac*_. Consequently, at the fixed value of the dc current *j*_*dc*_ < *j*_*th*_ the maximum ac current frequency ${f}_{ac}={f}_{1}^{th}$, at which the generation of the spike sequences is still possible, decreases with the increase of the effective damping *α*_*eff*_ of the AFM material (see Fig. 3(b)). We would like to note, that the effective damping *α*_*eff*_ can be adjusted not only by choosing a different AFM material having a different intrinsic damping *α*_0_, but also by changing the thickness of the AFM layer, as the spin-pumping-related part of the effective damping is inversely proportional to the AFM later thickness.

Note, that the maximum “clock” frequency of spike generation in the proposed artificial AFM “neuron” decreases with the increase of the effective damping, and is ultimately limited by the inverse duration of a single spike determined by Eq. (2). Our numerical simulations also show that this frequency can be increased up to *f*_*ac*_ > 150 GHz by the increase of the bias dc current (${j}_{dc}\simeq {j}_{dc}^{th}$) in a narrow range of the *α*_*eff*_ values. Nevertheless, the expected speed of the neuron operation is much greater then the biological counterparts and contemporary electronic processors.

A continuous generation of ultra-short pulses with a random initial phase is also possible in the absence of driving ac current by applying dc bias current with low supercriticality $0 < ({j}_{dc}-{j}_{dc}^{th})/{j}_{dc}^{th}\ll 1$, see right bottom corner in Fig. 3(a). The frequency of the pulse train in this case rapidly increases with the value of dc current^20^ as $f \sim \sqrt{{j}_{dc}-{j}_{dc}^{th}}$ and is not stable under the fluctuations of the dc bias. However, such regime of the spikes generation can be phase-locked by the injection of ac control signal, which is indicated by the bending of the single-spike phase for small values of *j*_*ac*_ in Fig. 3(a). This effect to be considered in detail elsewhere.

**Figure 5:**
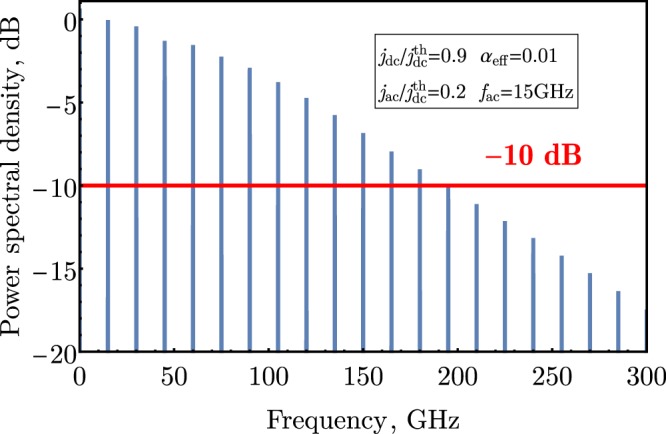
Numerically simulated frequency comb (spectrum of a periodic sequence of spikes) generated by an AFM SHO. Spectrum is normalized by the amplitude of the first harmonic at *f*_*ac*_ = 15 GHz.

The application of the proposed AFM generator of ultrashort spikes for traditional signal processing purposes, for example, as a spintronic frequency multiplexer, is also possible, and it requires a sufficiently wide frequency bandwidth of the generated signal. The simulated spectral density of a spike sequence generated by the above described AFM auto-oscillator at the ac driving frequency of *f*_*ac*_ = 15 GHz is shown on Fig. 5. The spectrum represents a well-known *frequency “comb”* with a slow decay of the amplitude of higher harmonics with the increase of the harmonic number. The generation bandwidth, which is, obviously, defined by the duration of the single spike, reaches the value of $-{\rm{\Delta }}f\simeq 200\,{\rm{GHz}}$ at −10 dB. This value can be tuned by the choice of the parameters of a particular AFM auto-oscillator. In particular, that can be done by tuning the thickness of the AFM layer, and, therefore, tuning the effective damping parameter *α*_*eff*_, or by choosing a different AFM materal having a proper value of the in-plane anisotropy (see Eq. (2)).

To use the proposed artificial AFM neurons in a neuromorphic network one will need to connect several of the AFM neurons in such a way, that the outputs of one (or many) of the artificial neurons are connected via synapses to the input of a different artificial neuron. Since the output signal of an AFM is produced in a form of an electrical voltage, one can use, for example, spintronic memristors to modulate the coupling (or synapse strength) between the neurons.

The detailed consideration of the possible operation of networks of AFM artificial neurons falls out of the scope of our current work. However here, as an example, we consider an elementary network of two AFM neurons connected via a tunable synapse. In our model we have two AFM neurons (“master” and “slave”) having the output the master connected to the input of the slave via a tunable amplifier. Both neurons are driven by the same sub-critical bias DC current. We model the connection between the neurons by a scalar coupling coefficient *ν*, meaning that the power transmitted into the slave neuron from the master neuron is related to the power stored in the master neuron.

**Figure 6:**
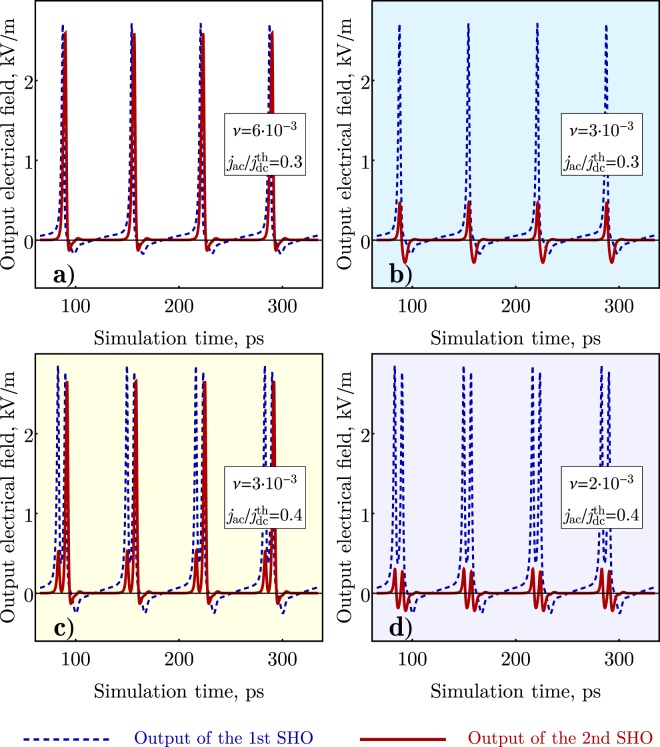
Numerical simulation of the operation of a network of two AFM SHO connected in a sequence for different values of the coupling coefficient between the SHOs and different modulation depths of a driving sinusoidal signal applied to the first (master) SHO.

The “master” neuron is driven by both sub-critical constant bias current and an additional sinusoidal stimulus. The typical response of a pair of coupled AFM neurons is shown in Fig. 6. If the coupling between the neurons is relatively strong, the “slave” neuron acts as repeater (Fig. 6(a)), but if the coupling is reduced, the “slave” neuron cannot fire Fig. 6(b) and remains “silent”.

If we increase the amplitude of the sinusoidal stimulus signal acting on the “master” neuron, and drive it into a bursting mode, the slave neuron can fire, but in a single pulse regime (Fig. 6(c)). Further reduction of the coupling coefficient can break the synaptic connection between the neurons (see Fig. 6(d)). In a realistic neuromorphic network the outputs of multiple “master” neurons could be connected to an input of a single slave neuron. In such a case, the reaction of the slave neuron would be a weighted response based on the multiple inputs - the slave neuron fires only once if the combined input exceeds a certain threshold threshold, which is very similar to the functionality of biological neural networks.

As a final remark, we would like to note, that the proposed mechanism of the AFM-based ultra-short spike generation is efficient for *relatively high values of the damping constant α*_*eff*_ ≥ 0.01. This means that metallic AFM materials, like Mn_2_Au or IrMn could be more suitable for the practical design of the AFM-based spike generators, than the dielectric AFM, like NiO. The use of conductive AFM layers in a spike generator can also substantially enhance the magnitude of the output signal by employing the “AFM tunneling magnetoresistance effect”^27,28^ instead of the ISHE in the adjacent Pt layer to extract the output spike signal from the AFM material.

## Methods

Most of the results were obtained by the numerical simulations of the spin dynamics in AFM, described by two coupled Landau-Lifshitz-Gilbert-Slonczewski equations for two magnetic sublattices **M**_1_ and **M**_2_ of the AFM, which were derived in^20^:

4 $$d{{\bf{M}}}_{i}/dt=\gamma [{{\bf{H}}}_{i}\times {{\bf{M}}}_{i}]+\frac{{\alpha }_{eff}}{{M}_{s}}[{{\bf{M}}}_{i}\times d{{\bf{M}}}_{i}/dt]+\frac{\sigma {j}_{e}(t)}{{M}_{s}}[{{\bf{M}}}_{i}\times [{{\bf{M}}}_{i}\times {\bf{p}}]],$$

where *j*_*e*_(*t*) is defined by Eq. (3), *i* = 1, 2 and **H**_1_ and **H**_2_ are the effective magnetic fields acting on the sublattices **M**_1_ and **M**_2_, respectively:

5a $${{\bf{H}}}_{1}=\frac{1}{{M}_{s}}[-\,\frac{1}{2}{H}_{{\rm{ex}}}{{\bf{M}}}_{2}+{H}_{h}{{\bf{n}}}_{h}({{\bf{n}}}_{h}\cdot {{\bf{M}}}_{1})-{H}_{e}{{\bf{n}}}_{e}({{\bf{n}}}_{e}\cdot {{\bf{M}}}_{1})],$$

5b $${{\bf{H}}}_{2}=\frac{1}{{M}_{s}}[-\,\frac{1}{2}{H}_{{\rm{ex}}}{{\bf{M}}}_{1}+{H}_{h}{{\bf{n}}}_{h}({{\bf{n}}}_{h}\cdot {{\bf{M}}}_{2})-{H}_{e}{{\bf{n}}}_{e}({{\bf{n}}}_{e}\cdot {{\bf{M}}}_{2})].$$

Here *H*_ex_ = *ω*_*ex*_/*γ* is the exchange field, *H*_*e*_ = *ω*_*e*_/*γ* and *H*_*h*_ = *ω*_*h*_/*γ* are the easy-plane and hard-axis anisotropy fields, respectively. It should be noted, that the variation of *H*_*h*_ value does not affect the results, see Eq. (1).

The time evolution of Eq. (4) was calculated for the domain 0 .. 100*T*, where *T* = 1/*f*_*ac*_ is the period of applied ac signal. The first 10 periods were cut off from the analysis to filter out any initial transient processes. Thereafter, the counted number of **M**_1_ and **M**_2_ flips–*n*_*sf*_ was compared with the number of the ac periods–*n*_*ac*_. The “Single spike” on Fig. 3 correspons to *n*_*sf*_/*n*_*ac*_ = 1, “No spikes”−*n*_*sf*_/*n*_*ac*_ < 1 and “Bursting”−*n*_*sf*_/*n*_*ac*_ > 1.

The output electric field, see Fig. 2, was calculated as *E* = *κ*$\dot{\varphi }$, where *κ* = 1.35 × 10^−9^ V/m⋅(rad/s)^−1^ was taken from^20^ as well as another parameters.
